# Supplementary material for: Early Feasibility of an Activity-Based Intervention for Improving Ingestive Functions in Older Adults with Oropharyngeal Dysphagia
Source: Geriatrics (Basel). 2023 Apr 19;8(2):44. doi: 10.3390/geriatrics8020044 (PMC10137850; doi:10.3390/geriatrics8020044)
Supplement: Supplementary file 1 [file geriatrics-08-00044-s001.zip › geriatrics-2274050-supplementary.pdf]

**Supplemental file Table S1: The intervention described according to the TIDieR (Template for Intervention Description and Replication) [1]**

**BRIEF NAME**

1. ***Provide the name or a phrase that describes the intervention.***

ACTivity-based strength- and skill-based training of swallowing to improve INGestion (ACT-ING program).

**WHY**

2. ***Describe any rationale, theory, or goal of the elements essential to the intervention.***

*Rationale for the intervention*:

Sarcopenic oropharyngeal dysphagia (OD) is difficulty in swallowing associated with loss of generalized skeletal muscles and swallowing-related muscles [2-4]. The prevalence of sarcopenic OD is estimated to be around 30% in acute care hospitals as well as rehabilitation settings [5]. The presence of sarcopenia and OD is complex and seems to generate detrimental synergies, where swallowing becomes gradually weaker, slower, and uncoordinated [2-4]. Sarcopenia in the muscles of the aerodigestive tract can be primary due to aging per se (i.e., presbyphagia), or secondary in the event of 1) activity-related factors, which include unnecessary inactivity or unnecessary nil per mouth, 2) nutrition-related factors, which include inappropriate nutritional care management, and 3) disease-related factors, which include polypharmacy or iatrogenic diseases [6]. In the acute hospital phase, activity- and nutrition- related factors are more likely to cause secondary (i.e., iatrogenic) sarcopenia than disease-related factors [7]. Activity-related factors such as being on a texture-modified diet (TMD) that is poorly accepted and adhered to or nil by mouth, decrease functional oral intake as well as swallowing frequency, which might result in inactivity-induced sarcopenia. In addition, TMD does not meet the participant's nutritional requirements, which might cause nutrition-related sarcopenia [8]. Therefore, the rehabilitation method for sarcopenic OD includes two aspects: nutrition care management in consideration of rehabilitation, and rehabilitation in consideration of nutrition [6]. These are addressed in the ACT-ING program [8].

*Goal of the intervention*:

The ACT-ING program aims to maximize swallowing-related outcomes and prevent further decline of the swallowing muscles in older adults at risk of or with sarcopenic OD [8].

*Theory for the intervention*:

The ACT-ING program uses goal-directed and task-specific swallowing exercises in eating and drinking activities to improve strength, speed, and coordination of swallowing [8]. The underlying theory is informed by a person-centered, task-oriented approach based on a systems model of motor behavior derived from motor learning theories [9]. In other words, the intervention integrates a person-centered focus and combines strength- and skill-based training principles [10] in purposeful tasks and activities to improve motor performance [8].

Strength training involves concentric and eccentric muscular contraction with the goal of improving muscular strength (the maximum amount of force a muscle or muscle group can exert), endurance (the ability of a muscle or a muscle group to exert force consistently and repetitively over a period), and power (the ability to generate as much force as quickly as possible). This is accomplished by manipulating the load and the number of repetitions, sets, tempo, exercises, and force to overload a group of muscles [11].

The skill-based training principles are classified into two types: (1) principles related to the structure of practice, and (2) principles related to the nature of feedback. The principles related to the structure of practice include conditions such as practice variability (constant vs. variable), practice distribution (massed vs. distributed), practice schedule (random vs. blocked), attentional focus (external vs. internal), and target complexity (simple vs. complex). Feedback conditions include type, frequency, and timing [10].

For the ACT-ING program, the concept of affordance [12] is utilized to explain the integration of strength- and skill-based training principles in the complex person-environment interaction during eating and drinking tasks/activities. Affordance refers to properties of the environment [12] (e.g., food and liquid items, which are characterized by multi-sensory and highly integrated properties like flavor, mouthfeel, texture and structure, temperature, shape, size, volume, and palatability) [8]. These properties can be detected as information to support an action, which is related to an individual's action capabilities [12]. Affordance is task specific and perceived by the performer to select an appropriate movement solution to achieve the goal of the task [12]. That is, the amount of force produced by the muscles in the mouth and throat is afforded by the perceived rheological properties of food and liquid items (e.g., thicker liquid consistencies and larger volumes elicit higher tongue pressure during swallow, and increased food hardness increases the number of chews and muscle recruitment levels of the masticatory muscles and the movements of the tongue during the oral processing) [8]. In addition, selectively attending to affordance information supports optimal motor behavior (e.g., by volitional modulation of oral and pharyngeal events by using swallowing maneuvers, such as effortful swallowing) [8].

The person-centered procedure of the intervention is informed by self-determination theory (SDT) [13], in which it is assumed that the quality of motivation experienced by individuals when engaging in, for example, the ACT-ING program, is determined by the extent to which they view their behaviors and actions to be consistent with three basic psychological needs: 1) autonomy (i.e., a feeling of ownership and responsibility, where actions are experienced as freely chosen and self-endorsed); 2) competence (i.e., a feeling of confidence and effectiveness in action); and 3) relatedness (i.e., a feeling of being accepted and respected and being socially connected and cared for). In SDT, the quality of motivation relates to the kind of motivation that drives the individual's behavior and action. Motivation is described as a continuum from 1) being a-motivated (i.e., lack of perceived competence, lack of value, or nonrelevance) through 2) extrinsic motivation (i.e., controlled behavior with perceived contingency between the behavior and a consequence such as a reward) to 3) intrinsic motivation (i.e., self-determined behavior with perceived competence, interest, enjoyment, and inherent satisfaction) [13]. To support intrinsic motivation and thus the three basic psychological needs, when delivering the ACT-ING program, a set of supportive strategies for providing feed-back during the intervention is specified in the intervention manual [8] and presented in the TIDIeR item 4.

The fundamental proposition of the ACT-ING program is that when the participant experiences fulfillment of the three basic psychological needs, this will promote autonomous intrinsic motivation for engaging in the therapeutic swallowing attempts, which will be reflected by in-therapy engagement, as well as improvements in perceived swallowing capacity during the course of the therapy. These putative mediators of change are expected to result in improved strength, speed, and coordination of the swallowing function (i.e., short-term outcomes), leading to improvements in ingestive skills and functional oral intake during meals (medium-term outcomes) which will minimize the severe complications associated with sarcopenic OD, such as malnutrition, dehydration, aspiration pneumonia, increased hospitalizations, increased mortality, and affected quality of life (long term outcomes). This is outlined in a logic model in Hansen et al. [8].

**WHAT**

3. ***Materials: Describe any physical or informational materials used in the intervention, including those provided to participants or used in intervention delivery or in training of intervention providers.***

3.1. Used in intervention delivery.

Mealtime equipment/utensils, thickener, and a syringe (10 ml) or measuring spoons (1-2-5-10 ml). Food and liquid items of different textures and volumes arranged in a 17-level task hierarchy with increasing resistive load related to demands for involvement and pressure generation of the ingestion musculature [8]. The task hierarchy is based on the International Dysphagia Diet Standardization Initiative (IDDSI) terminology and accompanying color codes, and is presented in the manual as shown in Table 1.

3.2. Intervention manual used by intervention providers.

A standardized intervention manual (in Danish) describing the intervention procedures and containing worksheets and training logs to be used by the interventionists. The manual consists of six main parts: 1) a brief introduction and description of the target group; 2) theory and general principles underlying the ACT-ING program; 3) suggestions for clinical assessments and procedure for goal-setting using SMART goals; 4) duration and frequency, training load using the task hierarchy and how to use it, description of the effortful swallowing technique for proper form and how to instruct participants, establishing start level, and control of effort and progression during therapy; 5) documentation of delivered therapy, including an example of a completed worksheet and training log; and 6) tips and problem-solving.

**Table 1. Task-hierarchy with increasingly resistive liquid and food-items to be used in the ACT-ING program**

| Level | Load as volume | Load as textures[a] | Actions |
|---|---|---|---|
| 1 | Saliva/1 ml | Effortful swallow using dry swallow or thin liquid. | Squeeze very hard with the tongue and throat muscles throughout the swallow. |
| **2-3-4** | 2 ml-5 ml-10 ml | **Mildly thick liquids:** oral supplements (protein-drink), cocoa milk, creamy soups without solid contents, juice (pineapple, prune, apricot, tomato), some shakes, soft ice cream, thickened liquids as per IDDSI. | Tongue controls and propels bolus backwards in the oral cavity for swallowing. Requires some propulsion effort. |
| **5-6-7** | 2 ml-5 ml-10 ml | **Moderately thick liquids/liquidized foods:** runny pureed food, fruit syrup, sauces and gravies, yogurt, thickened liquids as per. IDDSI. | Tongue controls and propels bolus backwards in the oral cavity for swallowing. Requires more propulsion effort than levels 2-4. |
| **8-9-10** | 2 ml-5 ml-10 ml | **Extremely thick liquids/pureed foods:** thick yoghurt/skyr, smooth porridge, purees (meat, fish, vegetables, pasta, fruit), pudding, mousses, thickened liquids as per. IDDSI. | Tongue controls and propels bolus backwards in the oral cavity for swallowing. Requires more propulsion effort than levels 5-7. |
| **11 -12** | 5 ml-10 ml | **Minced and moist:** thick and smooth porridge with small soft lumps, jelly bread, mashed boiled potatoes/vegetables/fruit (drain any liquid), finely minced or chopped/pureed meat /fish in thick, soft cohesive sauce or gravy. | Minimal chewing, tongue force alone can be used to break down soft small particles. Tongue force to form and move the bolus backwards in the oral cavity for chewing. Requires more propulsion effort than levels 8-10. |
| **13** | 1.5 cm x 1.5 cm | **Soft & bite-sized:** soft, tender, and moist with no separate thin liquid. E.g.: cooked tender meat/fish, casserole/stew/curry; steamed/boiled vegetables; cooked pasta or rice; eggs of any kind (not fried hard); yoghurt/skyr with soft fruit, soft cheese, porridge with small soft lumps, soft fruit, soft/moist cake, bread without grains and crusts. | Chewing, tongue force and control to form and move the bolus for chewing. Tongue force to move the bolus backwards in the oral cavity for swallowing. Requires more endurance and propulsion effort than levels 8-10. |
| **14** | Self-determined | **Easy to chew:** normal everyday foods of soft/tender textures. Food items as level 13 with self-determined bitesize. | As level 13, but requires more endurance and propulsion effort than levels 8-10. |
| **15** | Self-determined | **Regular:** crumbly (biscuits), crunchy (raw carrot), crispy (crispy bacon), hard or dry (nuts, raw carrot), fibrous or tough (steak, pineapple), stringy (beans), chewy (cheese chunks, dried fruits), sticky (nut butter, overcooked oatmeal), floppy (lettuce). | As levels 13-14, but requires more occlusal force, masticatory activity, force, endurance, and propulsion effort than level 14. |
| **16** | Self-determined | **Regular:** thin liquids (water, coffee/tea, milk, thin juice, sorbet/Italian ice cream), mixed thin-thick textures (soup with solid pieces), juicy food where the juice separates from the solid in the mouth (watermelon). [b] | Tongue control to balance liquids. When textures are mixed, the tongue separates the liquids from solids and controls/moves the bolus backwards in the oral cavity for swallowing. |
| **17** | Participant's goal | Participant's choice | Dependent on participant's choice |

Abbreviations: ACT-ING, ACTivity-based strength- and skill-based training of swallowing to improve INGestion; IDDSI, International Dysphagia Diet Standardization Initiative

[a] Examples of liquid and food-items are according to Danish food culture (not an exhaustive list).

[b] Thin liquids and juicy foods are introduced late as they require complex coordination of ingestion functions due to a very fast flow and may pose a risk of aspiration.

4. ***Procedures: Describe each of the procedures, activities, and/or processes used in the intervention, including any enabling or support activities.***

The procedures are related to 1) the intervention process in terms of supportive techniques and feedback and 2) the intervention procedure for the combined strength- and skill-based training.

*4.1 Intervention process*

Overall, the SDT-informed supportive strategies [8,13] (Table 2) and types of feedback [8,10,12] (Table 3) address: 1) goal-setting; 2) engagement in shared decision-making (e.g., the liquid and food items are selected in collaboration with the participants to ensure that the sensory perception and experience of the ingested liquid and food are pleasant, which will positively affect performance during training and vice versa); 3) how to provide verbal cues and feedback that are rich, clear and effectance-relevant; 4) how to ensure optimal challenges by gradually increasing (or decreasing) the exercise load; and 5) how to behave with unconditional respect, care and support, promoting a warm social environment and avoiding use of controlling pressure or incentives [8].

In the first therapy session, the participants receive information on dysphagia in old age and the purpose and content of the ACT-ING program, and they are involved in goal-setting, shared decision-making related to the frequency and duration of the therapy and self-training, and in identifying barriers to task performance and ways of overcoming them in order to strengthen motivation (e.g., how to protect the airway from aspiration and how to clean in case of aspiration). During the course of the therapy, the supportive strategies (Table 2) and types of feedback (Table 3) are tailored to each participant.

**Table 2. SDT-informed supportive strategies [8,13] in the ACT-ING program**

| |
|---|
| **Autonomy-supportive techniques** (i.e., the interventionist has unconditional regard and a desire for empowerment and self-actualization by embracing the participant's perspective). |
| **Take the internal frame of reference.** |
| • Acknowledge the participant's perspective. Take time to understand the perspective and recognize possible challenges. |
| • Explore the participant's expectations for participating in the ACT-ING program. |
| • Encourage identification of intrinsic goals. |
| **Provide a meaningful rationale for activities.** |
| • Provide rationales for undertaking the activity components of the ACT-ING program. |
| • Offer choice and variety that are realistic and relevant to the participants' needs (e.g., the participant is involved in the selection of liquid and food items from the task hierarchy). |
| • Relate the content of the ACT-ING program to meaningful life events (e.g., participation in meal related social activities). |
| **Acknowledge feelings of resistance.** |
| • Embrace the participant's perspective (e.g., emotional, interpersonal, or practical issues) and invite them to enter into a collaborative effort to overcome issues (e.g., by using some of the other SDT-based strategies). |
| **Provide choice and invite meaningful input.** |
| • Encourage the participant to take responsibility in decision-making and/or leadership (i.e., goal-setting using SMART goals related to eating and drinking activities; action planning related to the intensity of the therapy and self-training; instruction on how to exercise expected behavior during therapy and self-training). |
| • Facilitate active participation in decision-making related to structure (e.g., frequency of therapy sessions and amount of self-training, as well as input to structure therapy sessions). |
| • Encourage the participant to experiment and self-initiate ingestion of liquids and food items from the achieved task hierarchy level in-therapy when performing self-training in daily meals. |
| **Avoid the use of controlling pressure or incentives.** |
| • Use informational, non-judgmental language that conveys freedom of choice, collaboration, and possibility when communicating. In other words, avoid constraining, pressuring, or guilt-inducing language (e.g., use 'might' or 'could' instead of 'should' and 'must'). |
| **Need-supportive limit-setting.** |
| • Be clear about the limit, provide a meaningful rationale for its imposition, acknowledge and be emphatic about conflicts with or resistance to the limit, and provide choices (e.g., in the event of aspiration, explain why a lower level of the task hierarchy is recommended instead of a higher level, and involve the participant in choosing acceptable food items from the low levels). |

**Relatedness-supportive techniques** (i.e., the interventionist supports the participant's self and establishes rapport, alliance, and connection).

**Unconditional positive regard.**

- Demonstrate unconditional respect, care and support and promote a warm social environment.
- Show genuine appreciation and concern for the participant.
- Support the participant's active participation in planning and possible revision of goals and activities.
- Encourage the participant in achieving the planned therapy (i.e., planned numbers of repetitions or sets).

**Take an interest in the person.**

- Explore habits and preferences related to liquid and food items and meal performance.
- Provide statements of interest and curiosity about the participant's thoughts and perceptions, personal history and background, social context, life events, etc. when communicating.
- Pay continuous careful attention to, and gather knowledge about, the participant during assessment and therapy.
- Attend to every sign of pain or aspiration and react with appropriate supervision and support.
- Express a personal interest in the participant and take time to develop a rapport.

**Acknowledge/accept conflict.**

- Show unconditional regard expressing positive support regardless of success or failure.
- Provide statements of empathy and acknowledgment of the participant's perspective, conflicts/ambivalence, distress, and negative affect (fear, confusion, etc.) and express positive feelings when communicating with the participant (concerning task performance, therapy, or other related matters).
- Attend to and identify possible concerns or challenges of the participant and involve the participant in solutions (e.g., encourage the participant to seek social support from others or try out alternative ways of performance in case of challenges with self-training).

**Authenticity and transparency**

- Listen empathically by demonstrating attentiveness to the participant's responses (e.g., be silent to allow the person to complete sentences) and provide reflective and summary statements when appropriate (directed at affect or content) when communicating. Prompt permission to provide new information, guidance, or advice.
- Be honest when expressing concern, interest, or openness.
- Reflectively share important and meaningful perceptions and experiences with the participant without imposing on them.

**Competency-supportive techniques** and provision of structure (i.e., the interventionist provides feedback and guidance, graduates therapy appropriately, and provides clear expectations about outcomes of exercises).

**Identify barriers and obstacles.**

- Enable the participant to identify barriers to task performance and identify ways of overcoming them to strengthen motivation (e.g., how to protect the airway from aspiration and how to clean in case of aspiration).

**Focus on optimal challenges.**

- Foster clarity of expectations by collaboratively setting realistic goals and discussing what to expect and not expect from the ACT-ING program.
- Assist in identification of eating- and drinking-related goals that are realistic, meaningful, challenging, and achievable.
- Give proper instruction in performing effortful swallowing, and repeat if necessary, during therapy.
- Provide optimal challenge-matching/tailoring of the hierarchical task level to the participant's skills.

**Promote an internal rather than an external perceived locus of evaluation.**

- Promote monitoring of progress, skill level, and performance by using the RPE scale, food diary, including steps toward the participant's goal.
- Develop and provide a summary of therapy sessions to work toward how to integrate self-training into daily meals.

**Offer rich, clear, and effectance-relevant feedback.**

- Provide general support and encouragement (i.e., social support from the interventionist) during exercises (e.g., verbal encouragement about capability, affirmation, support for change/persistence, and support in how to integrate therapy into daily meals).
- Facilitate focus on completing the process of the task, matched against the participant's own standards, rather than the outcomes of the task.
- Provide informational feedback giving information on how the participant achieved/did not achieve a desired outcome during task performance, rather than generic praise/criticism.

- Provide continual information on the content and expected outcomes of the ACT-ING program to participants relevant to their needs and situation.

**Encourage reflective consideration of consequences.**

- Encourage the participant to reflect on cost (e.g., effort, burden) and benefit (e.g., progress) during therapy.
- Encourage the participant to reflect on how to transfer exercises during therapy to self-training in daily meals.

The competency-supportive techniques are also used when the interventionist provides feedback as part of the skill-based training principles. The principles relating to the nature of feedback are shown in Table 3.

Table 3. Types of feed-back [10,12] during the ACT-ING program

| Types of feedback | Initial level | More trained level |
|---|---|---|
| Type of errors | It is expected that the participant might compensate or aspirate during progression. If the participant can clear the throat efficiently, aspiration in 20% of the swallow repetitions in a set is regarded as acceptable. | It is expected that the participant will compensate or aspirate less frequently during progression. |
| Focus (knowledge of performance or knowledge of results) | The participant receives feedback on performance in terms of whether they performed the effortful swallow adequately (knowledge of performance). | The participant receives feedback on whether they swallowed successfully (efficiently and safely) (knowledge of result). |
| Frequency (high frequency or Low frequency) | The participant receives feedback after each swallowing repetition. | Participant receives feedback after each set of swallowing repetitions. |
| Timing (immediate or delayed) | The participant is given feedback immediately after each swallowing repetition. | The participant is given delayed feedback a few seconds after a set of swallowing repetitions is completed. |
| Control (external or internal) | The therapist provides feedback on the result/outcome. | The therapist encourages the participant to be aware of the information from their own motor system to help self-evaluate the swallowing performance. |

*4.2 Intervention procedure*

The intervention procedures involve learning form and technique, establishing the training start level, performance of the therapeutic swallowing attempts, control of effort, and progression criteria.

4.2.1. Form and technique

The starting position is in an upright aligned sitting position with the head held in a neutral and slightly flexed position. If needed, modification and support of body position is given. Initially, participants practice the exercise technique of effortful swallowing, where the muscular effort during a typical bolus swallow is consciously increased, which results in increased movements of the oropharyngeal structures and pressure generation in the muscles of the tongue and pharynx [15]. Participants are instructed to place the tip of the tongue on the palate behind the upper teeth and swallow as forcefully as possible while pushing hard with the tongue and squeezing the neck muscles (i.e., task hierarchy level 1 in Table 1). Effortful swallowing is confirmed by the interventionist through visual observation of facial expression, contraction of the orofacial and suprahyoid muscles, and palpation of the suprahyoid muscles and larynx using Logemann's four-finger test to confirm laryngeal elevation [8].

4.2.2. Establishing the training start level

To ensure that initial training is of sufficient intensity to cause an increase in muscle strength while at the same time ensuring that the swallow is safe and without aspiration, a way of quantifying the load called the functional repetition maximum (FRM) is used [8,11]. The repetition maximum is defined as the maximum load from the task-hierarchy that permits five swallowing repetitions without aspiration. When effortful swallowing (task level 1) is mastered, participants start test swallowing attempts with five effortful swallowing repetitions with food and liquid items, starting with level 2 (2 ml of mildly thick liquid) and continuing to level 10 (10 ml of extremely thick liquid/pureed food) [8]. A

successful swallowing test means that the participant can manage a specific level of the task hierarchy five times without clinical signs of aspiration (coughing, gurgling breathing/wet voice, or breathing difficulties). The choice of FRM is based on assessment of the participant's ability using the score sheet in Table 4.

**Table 4. Establishing the functional repetition maximum (FRM)**

| Level | Load | Mark the absence or presence of clinical signs of aspiration for each of five effortful swallows.<br><br>√= No clinical sign of aspiration<br>O= Clinical sign of aspiration | | |
|---|---|---|---|---|
| | | **Coughing** | **Gurgling breathing/wet voice** | **Breathing difficulties** |
| **Mildly thick liquids** | | | | |
| **2** | 2 ml | 1 2 3 4 5 | 1 2 3 4 5 | 1 2 3 4 5 |
| **3** | 5 ml | 1 2 3 4 5 | 1 2 3 4 5 | 1 2 3 4 5 |
| **4** | 10 ml | 1 2 3 4 5 | 1 2 3 4 5 | 1 2 3 4 5 |
| **Moderately thick liquids/liquidized foods** | | | | |
| **5** | 2 ml | 1 2 3 4 5 | 1 2 3 4 5 | 1 2 3 4 5 |
| **6** | 5 ml | 1 2 3 4 5 | 1 2 3 4 5 | 1 2 3 4 5 |
| **7** | 10 ml | 1 2 3 4 5 | 1 2 3 4 5 | 1 2 3 4 5 |
| **Extremely thick liquids/pureed foods** | | | | |
| **8** | 2 ml | 1 2 3 4 5 | 1 2 3 4 5 | 1 2 3 4 5 |
| **9** | 5 ml | 1 2 3 4 5 | 1 2 3 4 5 | 1 2 3 4 5 |
| **10** | 10 ml | 1 2 3 4 5 | 1 2 3 4 5 | 1 2 3 4 5 |

### 4.2.3. Control of effort and progression

Once the start level has been established, the participant begins with therapeutic swallowing attempts at that specific task level using effortful swallowing. At least two different liquid or food items within a level of the task hierarchy are used alternately to increase sensory stimulation and practice variation [8]. The minimum number of repetitions for each liquid or food item will be five, which is progressed during therapy as described below and in Table 5.

To prevent the intensity being too high or too low, control of effort is undertaken by using a rating of perceived exertion (RPE) scale from 0 (extremely easy) to 10 (extremely hard) with visual illustrations (an OMNI-RES scale) (Fig.1) [16]. After each set of swallowing repetitions, the participants are asked to use any number on the scale to rate their overall muscular effort, and the interventionist uses the same wording, 'How hard do you feel your muscles in your mouth and throat were working when you swallowed?'… 'A rating of 0 is associated with no effort (rest), and a rating of 10 is associated with the most stressful swallow ever performed'.

**Figure 1.** Rating of perceived exertion scale.

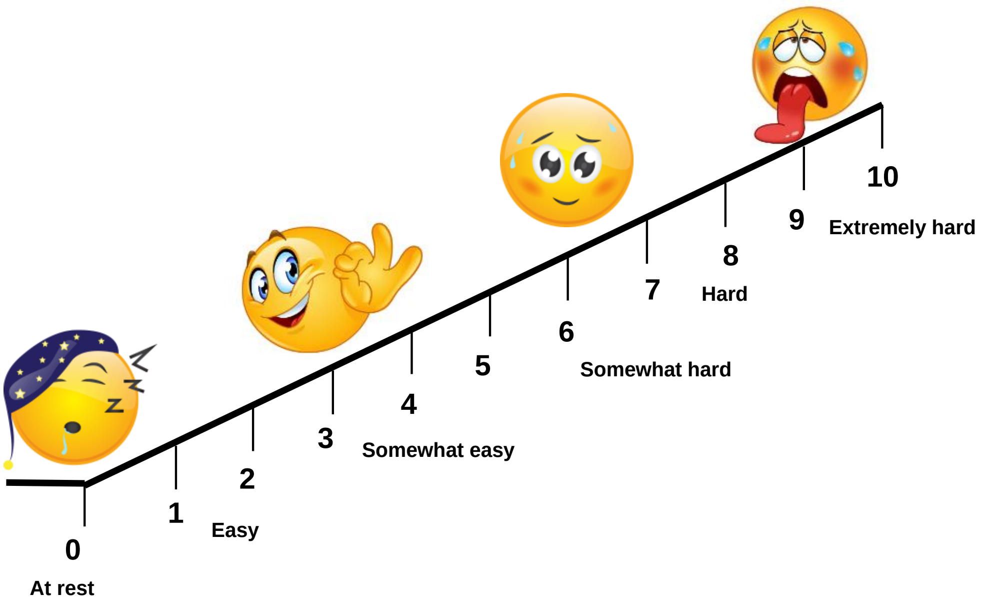

As therapy progresses, advancing steps of altered bolus volume, bolus consistency, and swallowing repetitions are introduced according to the 17-level task hierarchy in Table 1. This is based on predetermined progression rules:

- Progression within a level of the task hierarchy and according to the three characteristics of muscular fitness is realized by altering intensity variables in terms of practice variability (training load/material, tempo), practice amount (number of swallows across sets), and practice distribution (number of sets and rest periods), as outlined in Table 5.
- Progression across the task hierarchy is achieved when the participant can perform three sets of 12 swallowing repetitions without clinical signs of aspiration and with a decrease in RPE.

**Table 5. Exercise intensity variables included in the ACT-ING program**

| | Muscular strength (untrained / trained) | Muscular endurance (untrained / trained) | Muscular power (untrained / trained) |
|---|---|---|---|
| **Practice variability** | | | |
| Load | 5RM / achieved task level | One level below muscular strength | One level below muscular strength |
| Swallowing speed/tempo | Slow-moderate / moderate | Slow / moderate-fast | Moderate-fast / fast |
| **Practice amount** | | | |
| Swallowing repetitions | 5-8 / 8-12 | 10-15 / 15-20 | 3-6 / 3-6 |
| **Practice distribution** | | | |
| Sets | 1-3 / 4-6 | 1-3 / 4-6 | 1-3 / 4-6 |
| Rest period between sets | 1-3 min | ½-1 min | 1-3 min |
| **Control of effort** | | | |
| RPE scale | 5-6 / 7-8 | 3-4 / 5-6 | 3-4 / 5-6 |

## WHO PROVIDED

5. ***For each category of intervention provider describe their expertise, background and any specific training given.***

Occupational therapists (OTs) who had at least one year of postgraduate dysphagia management experience and postgraduate education in dysphagia management. Three OTs were affiliated to the physiotherapy and occupational therapy department at the hospital and three OTs were affiliated to two municipalities in the uptake area of the hospital.

It was planned that participating OTs should attend a one-day training course on delivering and adjusting the ACT-ING program for different severity levels of swallowing ability, sensation, and cognition, and how to manage self-directed practice and record the content and amount of therapy provided to the participants in the home, and in outpatient, and inpatient settings. Due to the COVID-19 prevention actions, it was not possible to carry out the planned training for the participating OTs. Instead, they received the intervention manual for preparation, were given a one hour individual introduction by the principal investigator (T.H.), observed therapy sessions delivered by T.H., and had responsibility for delivering therapy sessions when T.H. was unavailable. If needed, the OTs consulted T.H. and were given supervision to ensure consistency of intervention delivery.

## HOW

6. ***Describe the modes of delivery (e.g. face-to-face or by some other mechanism, such as internet or telephone) of the intervention and whether it was provided individually or in a group.***

Therapy is provided individually and is delivered face-to-face.
Between therapy sessions, the participants perform self-training in daily meals and document this in a food diary.

**WHERE**

7. ***Describe the type(s) of location(s) where the intervention occurred, including any necessary infrastructure or relevant features.***

The ACT-ING program was delivered in an inpatient (on the ward) or outpatient hospital setting (in the occupational therapy department), or in community-based rehabilitation centers or participants' homes.

**WHEN and HOW MUCH**

8. ***Describe the number of times the intervention was delivered and over what period of time including the number of sessions, their schedule, and their duration, intensity or dose.***

The ACT-ING program is intended to be delivered 2-3 days a week for up to 12 weeks with up to 45 minutes per therapy session. Thus, the maximum possible dose is 36 therapy sessions, or up to 27 hours for any participant. The intervention can be completed before the end of the 12 weeks if the participants achieve their goal or reache a sufficient level of functional oral intake (i.e., a total oral diet of multiple consistencies without special preparation, but with few specific avoidances or limitations (FOIS score 6 [17]).

**TAILORING**

9. ***If the intervention was planned to be personalized, titrated, or adapted, then describe what, why, when, and how.***

The intervention manual details procedures for tailoring practice activities according to the level of the participants' skills. The initial level of load/resistance is tailored to each participant as described in Item 4. 2.2. Likewise, progression is tailored to each participant as described in Item 4.2.3.

**MODIFICATIONS**

10. ***If the intervention was modified during the course of the study, describe the changes (what, why, when, and how).***

**Item 3 / Materials:** Development of a portable toolkit of different training materials (e.g., small puddings and mousses, soft and hard fruits and vegetables, dried fruits, biscuits and crackers, nuts, candies). Developed due to limited access to a varying range of food and drink items in all settings, which impacted the implementation of the task hierarchy. The toolkit supplied the participants' own meal items if needed.

**Item 4 / Intervention procedure**

4.2.2. Establishing the training start level

Modified early in the feasibility study to improve fit with the participants' needs and swallowing ability level. The modifications involved adjusting the FRM of five swallowing repetitions (5RM) to three swallowing repetitions (3RM). In addition, the loads were extended from levels 2-10 to levels 2-13 from the task hierarchy (Table 1). The criteria for determining the training start level were maintained.

4.2.3. Control of effort and progression

Progression within a level of the task hierarchy was modified early in the feasibility study to fit both the participants and the interventionists. The participating OTs perceived it to be too complex to administer the intensity variables for

the three trainable characteristics of muscular fitness (muscular strength, endurance, and power) in Table 5 and the accompanying worksheets (one worksheet per trainable characteristic of muscular fitness).

The modifications involved:

- Progression is implemented by altering the different practice conditions in terms of practice amount, distribution, variability, schedule, and complexity [10], which are outlined according to the ACT-ING program in Table 6. Using these principles, ensures that the intensity variables for different muscular finesses in terms of load, swallowing repetitions, speed/tempo, sets, and rest intervals are integrated indirectly into the therapy.
- The information on participants' in-therapy engagement during the therapy sessions is recorded on one accompanying worksheet covering: training materials (loads), sets, successful swallowing repetitions, signs of aspirations, signs of compensation, and control of effort.

Progression across the task hierarchy was also modified early in the feasibility study to fit with the participants needs and swallowing ability level. The modifications involved simplifying progression to a higher level of the task hierarchy to '*participant can perform one set of 10 swallowing repetitions without clinical signs of aspiration and with a decrease in self-perceived physical exertion using the RPE-scale*'. Regression from an earlier achieved level to a lower level of the task hierarchy is implemented if the participant shows clinical signs of aspiration in 20% of swallowing repetitions (i.e., 2/10 swallows or 1/5 swallows).

**Table 6.** Principles guiding the ACT-ING program

| Intensity variables and practice conditions | **Initial level** | **More trained level** |
|---|---|---|
| **Task-hierarchy** (load) | Task-levels 1.to 12. | Task levels 2. to 17. |
| **Practice amount** (swallowing repetitions) (small vs large) | The participant starts with 3-10 swallows when learning the effortful swallowing technique. | The participant completes more and more swallowing repetitions in a single session. Progression is realized by encouraging the participant to complete more swallow repetitions than in the previous session. The goal is > 50 swallowing repetitions per session. |
| **Practice distribution** (number of sets) (massed vs distributed) | The participant completes swallowing repetitions in a small period of time (one set) of 3-10 therapeutic swallowing without rest. | The participant completes swallowing repetitions distributed in sets of 3-15 swallow repetitions over longer sessions with a ½-1 minute rest between each set.<br>The progression is realized by increasing the number of sets as the participant progress in the number of swallowing repetitions. The goal is > 10 sets. |
| **Practice variability** (different muscles fit characteristics (strength, endurance, and power)). Periodization (constant vs variable) | The participant performs swallowing repetitions with the same training material (load) from the task hierarchy and swallows with a slow-moderate tempo. | The participant performs swallowing repetitions with different training materials (loads), different kinds of food or liquid items of the same load, and swallows with different speed/tempo (slow/moderate/fast) depending on the training material and/or encouragement by the therapist.<br>When the participant progresses in training material (load), then the easier levels from the task-hierarchy are retained to ensure variation.<br>As the therapy progresses, this will approach a natural meal and contribute to development of endurance of the swallowing musculature. |
| **Practice schedule** (blocked vs random) | The participant first completes a set of fast swallows with an easy level from the task hierarchy (e.g., level 3.: 10 ml of mildly thick liquids) and then completes a set of slow swallows with a more difficult level (e.g., level 9.: 5 ml of extremely thick liquids /pureed foods). | The participant performs the swallowing repetitions by intermixing different swallow dynamics (training materials, speed, timing) (e.g., one set is performed with two slow swallows of level 9. / One fast swallow of level 3. / Four slow swallows of level 9. / Two fast swallows of level 3. /One fast swallow of level 9. |
| **Task complexity** (simple vs complex) | The participant practices effortful swallowing with emphasis on tongue-to-palate contact. | The participant practices the whole movement of bolus control, formation and swallowing when progressing along the task hierarchy. |
| **Practice attentional focus** (internal vs external) | The participant focuses on body part in terms of how the tongue and larynx are moving during the effortful swallow. | The participant focuses on the effect of movement in terms of how well the training material (liquid /food) was swallowed and with what degree of physical exertion. |
| **Control of effort** using rating of perceived exertion (RPE) scale from 0-10 | The participant rates the RPE in the area of 5-6 /7-8 (somewhat hard/hard). | The participant rates the RPE in the area of 3-4/5-6 /7-8 (somewhat easy /somewhat hard / hard) across a range of practice conditions. |

**Item 6 / HOW**

Documentation of self-training using a food diary

Modified early in the feasibility study to improve fit with the needs of the participants, who experienced the food diary as burdensome without a clear purpose. The food diary for self-training was replaced by informal conversations on how to apply the exercises in daily meals and how to overcome any challenges.

---

**HOW WELL**

11. ***Planned: If intervention adherence or fidelity was assessed, describe how and by whom, and if any strategies were used to maintain or improve fidelity, describe them.***

The amount of the ACT-ING program for each participant was recorded on a specific worksheet as reported in the article 'Early feasibility of an activity-based intervention for strengthening ingestive functions in older adults with oropharyngeal dysphagia', for which this supplemental file is additional information.

12. ***Actual: If intervention adherence or fidelity was assessed, describe the extent to which the intervention was delivered as planned.***

The amount of the ACT-ING program for each participant is reported in the article 'Early feasibility of an activity-based intervention for strengthening ingestive functions in older adults with oropharyngeal dysphagia', for which this supplemental file is additional information. The factors reducing the maximum dose included participant illness, bank holidays, scheduling challenges, and staff absence.

**References (appear also in the original reference list, but with different numbers).**


1. Hoffmann, T.C.; Glasziou, P.P.; Boutron, I.; Milne, R.; Perera, R.; Moher, D.; Altman, D.G.; Barbour, V.; Macdonald, H.; Johnston, M.; et al. Better reporting of interventions: template for intervention description and replication (TIDieR) checklist and guide. BMJ (Clinical research ed.) 2014, 348, g1687. doi:10.1136/bmj.g1687.
2. de Sire, A.; Ferrillo, M.; Lippi, L.; Agostini, F.; de Sire, R.; Ferrara, P.E.; Raguso, G.; Riso, S.; Roccuzzo, A.; Ronconi, G.; et al. Sarcopenic Dysphagia, Malnutrition, and Oral Frailty in Elderly: A Comprehensive Review. *Nutrients* **2022**, *14*, 982. doi:10.3390/nu14050982.
3. Wakabayashi, H.; Kishima, M.: Itoda, M.; Fujishima, I.; Kunieda, K.; Ohno, T.; Shigematsu, T.; Oshima, F.; Mori, T.; Ogawa, N.; et al. Diagnosis and Treatment of Sarcopenic Dysphagia: A Scoping Review. *Dysphagia* **2021**, *36*, 523–531. doi:10.1007/s00455-021-10266-8.
4. Rajati, F.; Ahmadi, N.; Naghibzadeh, Z.A.; Kazeminia, M. The global prevalence of oropharyngeal dysphagia in different populations: a systematic review and meta-analysis. *J Transl Med* **2022,** *20*, 175. doi:10.1186/s12967-022-03380-0.
5. Nagano, A.; Ueshima, J.; Tsutsumichi, K.; Inoue, T.; Shimizu, A.; Mori, N.; Maeda, K. Effect of tongue strength on clinical outcomes of patients: A systematic review. *Arch Gerontol Geriatr* 2**022**, 102, 104749. doi:10.1016/j.archger.2022.104749.
6. Mizuno, S.; Wakabayashi, H.; Wada, F. Rehabilitation nutrition for individuals with frailty, disability, sarcopenic dysphagia, or sarcopenic respiratory disability. *Curr Opin Clin Nutr Metab Care* **2022**, 25, 29-36. doi: 10.1097/MCO.0000000000000787.
7. Dibello, V.; Zupo, R.; Sardone, R.; Lozupone, M.; Castellana, F.; Dibello, A.; Daniele, A.; De Pergola, G.; Bortone, I.; Lampignano, L.; et al. Oral frailty and its determinants in older age: a systematic review. *Lancet Healthy Longev* **2021,** *2*, e507-e520. doi:10.1016/S2666-7568(21)00143-4.
8. Hansen, T.; Thomassen, J.D.; Jensen, L.E.; Irgens, M.R.; Kjaersgaard, A. Development of an Intervention for Improving Ingestion in Elders with Oropharyngeal Dysphagia. *Phys Occup Ther Geriatr* **2021**, 39, 70-95.
9. Hosseini, S.A.; Ghamari, N.; Hayatizadeh, Z. Review of task-oriented interventions in occupational therapy. *J Rehab Med* **2017**, 6, 239–248.
10. Zimmerman, E.; Carnaby, G.; Lazarus, C.L.; Malandraki, G.A. Motor learning, neuroplasticity, and strength and skill training: moving from compensation to retraining in behavioral management of dysphagia. *Am J Speech Lang Pathol* **2020**, 29, 1065–1077. doi:10.1044/2019_AJSLP-19-00088.
11. Krekeler, B.N.; Rowe, L.M.; Connor, N.P. Dose in Exercise-Based Dysphagia Therapies: A Scoping Review. *Dysphagia* **2021**, 36, 1–32. doi:10.1007/s00455-020-10104-3.
12. Mathiowetz B. Task oriented approach to stroke rehabilitation. In *Stroke Rehabilitation: A Function-Based Approach*. 4th ed.; G. Gillen Ed.; Elsevier Inc, St. Louis, MD/USA, 2016, pp. 59–78.
13. Ryan, R.M.; Deci, E.L. S*elf-Determination Theory: Basic Psychological Needs in Motivation Development and Wellness*. 1st ed.; Guilford Press, New York, NY/USA, 2017.
14. The International Dysphagia Diet Standardisation Initiative (IDDSI). Complete IDDSI Framework: Detailed definitions, version 2.0. Published 2019. Available online: https://ftp.iddsi.org/Documents/Complete_IDDSI_Framework_Final_31July2019.pdf. (accessed on 7 August, 2019).
15. Bahia, M.M.; Lowell, S.Y. A Systematic Review of the Physiological Effects of the Effortful Swallow Maneuver in Adults With Normal and Disordered Swallowing. *Am J Speech Lang Pathol* **2020**, 29,1655-1673. doi.org/10.1044/2020_AJSLP-19-00132.
16. Lagally, K.M.; Robertson, R.J. Construct validity of the OMNI resistance exercise scale. *J Strength Cond Res* **2006**, 20, 252-256. doi:10.1519/R-17224.1.
17. Crary, M.A.; Mann, G.D.; Groher, M.E. Initial Psychometric Assessment of a Functional Oral intake Scale for Dysphagia in Stroke Patients. *Arch Phys Med Rehabil* **2005**, 86, 1516-1520.
